# The moderating effect of spiritual beliefs on job dissatisfaction related to the futile care

**DOI:** 10.1186/s12912-021-00582-7

**Published:** 2021-04-21

**Authors:** Farshid Alazmani-Noodeh, Kamel Abdi, Hadi Ranjbar

**Affiliations:** 1 School of Nursing and Midwifery, Iran University of Medical Sciences, Tehran, Iran; 2 Nursing Department, Faculty of Medicine, Komar University of Science and Technology, Sulaymaniyah, Kurdistan Region Iraq; 3 Institute for Futures Studies In Health, Kerman University of Medical Sciences, Kerman, Iran

**Keywords:** Nurse, Care, Futility, Spirituality, Job satisfaction, Critical

## Abstract

**Introduction:**

This study aimed to assess the experience of providing futile care among intensive care unit nurses and to examine the moderating effect of spiritual beliefs on job dissatisfaction related to the sense of futile care among nurses in Intensive Care Units.

**Materials and methods:**

The study had two phases. The first phase was a qualitative study. Twenty-two semi-structured interviews were conducted. In the second phase, we employed a cross-sectional design. The data from 236 nurses were collected using nurses’ perceptions of futile care questionnaire, Minnesota Satisfaction Questionnaire, and Spirituality and Spiritual Care Rating Scale.

**Results:**

The main theme of the qualitative phase was a feeling of self as a useful tool in God’s hand. Sub-themes were providing care while knowing it is futile, not knowing the patient destiny, having hope for care to be fruitful, experiences patient recovery, acting to be a part of God’s plan. Futile care and job experience were two predictors of low job satisfaction. Spiritual well-being had a moderating effect and increased job satisfaction.

**Conclusions:**

Futile care can decrease job satisfaction, while spiritual well-being can reduce its negative effect. Supporting spiritual aspects of nursing care can decrease turn-over intention among nurses.

## Introduction

The name of the Intensive Care Unit (ICU) is tied with futile care [1]. A large body of evidence linked the care in ICU to the sense of futility in nurses [1–4]. Futile care is defined as care that fails to provide clinical benefits [5]. However, there are various definitions of futile care in the literature which shows there is no unique approach toward it [6]. As a nursing teacher who worked as a nurse for 6 years in ICU, I never considered my care as futile, while I thought it was not successful. When I shared my point of view with some of my colleagues, I found that there is a big variation among my coworkers’ experiences regarding futile care. They had different experiences which lead to doing this study to examine nurses’ experience of providing futile care in the ICU.

Some studies have shown that futile care consumes a large number of hospitals’ resources and the spending sources for dying patients were higher than patients who have had a chance of survival [7, 8]. But the impact of futile care on healthcare providers especially nurses is more problematic than its other effects. Futile care is the subject of interest in many studies, especially ones that focused on health care providers’ burnout and job satisfaction [9–12].

Job satisfaction is defined as the global feeling or the general attitude of an individual toward his/her work [13]. It is reported to be associated with several variables such as the conflict between work and family, job demands, social support, and turnover intention [14, 15]. The results of some studies reported that job satisfaction is negatively associated with turnover among nurses [16–18]. Low job satisfaction is the most common cause of nurses’ turnover and intention to leave [19–21].

Spiritual well-being is the satisfaction with one’s sense of meaning and purpose in life and his/her relationship with a higher power or God [22]. It is also defined as people’s perception of the quality of their spiritual life [23]. Lee and Salman [24] defined Spiritual well-being as *“one’s perception regarding seeking the congruent, intrinsic, meaningful purpose of life and self-confidence to overcome challenges and achieve life goals”.* Spiritual care is defined as activities and interventions that promote spiritual health and the spiritual dimension of quality of life [25]. Spiritual well-being plays a significant role in nursing. Some studies showed that spiritual well-being is related to a better quality of life, job satisfaction [26, 27]. The relationship between the experience of providing futile care, job satisfaction, and spiritual well-being has remained unassessed. Also, how spiritual well-being can play a role in the sense of futile care and job satisfaction was another question that was raised and answered during this study.

### Study aims

This study had two phases. In the first phase, we assessed the experience of intensive care unit nurses of providing futile care. The second phase aimed to examine the moderating effect of spiritual beliefs on job dissatisfaction related to the sense of futile care among nurses in Intensive Care Units.

## Methods

This was a descriptive study that utilized a mixed-method approach. The study consisted of two phases. The first phase was a qualitative study in which we examined the experience of providing futile care among intensive care unit nurses. The second phase was a descriptive correlative study. The second phase was conducted to answer a question that was aroused from the first phase. The question was whether there is a relationship between spiritual wellbeing and the job satisfaction of nurses. Also, if there is a mediation effect of spirituality in the relationship between the sense of futile care and job satisfaction.

### Phase one

The first phase of the study was conducted using a qualitative approach. We interviewed 22 nurses. The demographic characteristics of nurses who participated in the qualitative study are presented in Table 1.

**Table 1:** demographic characteristics of nurses who participated in the qualitative study

	Age	Degree	Job Experience (Years)	Work in ICU (years)	Job Satisfaction
1	42	BSc	20	18	High
2	37	MSc	15	13	Moderate
3	39	BSC	14	10	Low
4	33	MSc	10	6	High
5	28	BSC	6	4	High
6	26	BSC	4	3	Low
7	27	BSC	6	6	High
8	29	BSC	7	7	Low
9	46	BSC	24	20	Low
10	44	BSC	23	19	High
11	32	MSc	12	10	Moderate
12	22	BSC	1	1	Low
13	23	BSC	2	2	High
14	25	BSC	3	3	Moderate
15	27	BSC	4	4	Low
16	29	BSC	5	4	High
17	28	MSc	7	6	Moderate
18	23	BSC	1	1	Low
19	24	BSC	2	2	Moderate
20	25	BSC	4	3	Moderate
21	32	BSC	9	6	Low
22	28	BSC	6	5	High
Mean	30.40		8.40	6.95	
Standard Deviation	7.021		6.87	5.744	

#### Samples and setting

The participants were recruited from intensive care units of three university-affiliated hospitals in Kerman, Iran. Inclusion criteria were having a nursing degree (at least BSc), and work as a practitioner in the intensive care unit. Because the relationship between providing futile care and low job satisfaction was mentioned in several studies, we used job satisfaction as an inclusion criterion. To find eligible key informants we distribute the Minnesota Satisfaction Questionnaire (MSQ among nurses of intensive care units along with scoring guidelines [28, 29]. Minnesota Satisfaction Questionnaire (MSQ) is fully discussed in the second phase of the study. We provided a phone number with a questionnaire, aim, and procedure of the qualitative phase and asked nurses who want to participate in the study to contact us. Among nurses who contacted us, we chose nurses based on their satisfaction scores. We divided the scores into four quartiles and selected nurses from each quartile. The summary of the characteristics of study participants is presented in Table 1. After sixteen interviews (Four interviews in each quartile), all existing sub-themes and their properties were identified, and no new sub-theme was formed. Four more interviews were conducted to ensure reaching data saturation.

#### Data gathering

Semi-structured in-depth interviews were the main method of data gathering. Each participant was invited by the first author (Ph.D. candidate in nursing) to the study in person. The time and place of the interviews were determined by participants and the first author. The first interviews were conducted based on an interview guide which was developed by the research team. The research team consisted of a nursing Ph.D. candidate with 10 years of experience as a clinical nurse and teacher, one nurse who is an expert in qualitative research, and an MSc in nursing who has experience of work in intensive care units. The primary interview guide consisted of three types of questions, including open general questions, intermediate and ending questions. Open general questions were used for the beginning of the interview, for example, “which cares do you consider as futile” or “describe your experience of providing futile care?”). The interviews were followed with intermediate questions such as “how did you feel when you provide cares that you felt are futile?”, “Why do you think your work is fruitful?” and “which factors influenced your feeling?”. Interviews were concluded by ending questions such as “Do you have anything to add?” or “is there something which you did not think about before this interview?”. Following and probe questions such as “can you give me an example?” and “can you explain more?” were used to clarify the statements of interviewees. The interview guide changed according to the data analysis, and new questions have emerged. Interviews last between 49 and 62 min. Each interview was listened to several times by the first author and then transcribed by him.

#### Data analysis

Data were analyzed using the thematic analysis approach developed by Braun and Clarke [30]. They introduced thematic analysis as a method for identifying, analyzing, and reporting patterns (themes) within data. In the first phase, the first-author familiarized himself with the data through immersion by repeated reading of interviews’ transcriptions and searching for meanings. The second phase was generating initial codes. An initial list of ideas about meaning units (whole interview, paragraphs, or sentences) was formed and each idea was labeled by a code. In the next step, initial codes were developed by the third author. Code labels were discussed in regular meetings by research team members. The third phase was searching for themes. In this phase, potential themes were formed by sorting and connecting relevant codes. Tentative themes were formed by the third author and discussed by all authors in meetings. In the fourth phase, tentative themes were refined and formed the main theme with four sub-themes. Sub-themes and the main theme were named in the fifth phase. Names were discussed and changed several times in research team meetings to reach an agreement over them. The final report was developed in the sixth phase. Representative codes and quotations were chosen to include in the article.

#### Rigor and trustworthiness

Suggested criteria for trustworthiness by Lincoln and Guba were used [31, 32]. To achieve credibility, prolonged engagement was used through immersion in data, dedicating enough time for data gathering and analysis, constant comparison of data with data, data with codes, codes with themes, and themes with each other. Member checks and peer checks were used to increasing dependability. Transcribed interviews and codes were checked with participants. The final theme and sub-themes were discussed with two participants. Coding, finding patterns, and naming the main theme and sub-themes were conducted in team sessions. We limited the initial literature review to decrease the effect of preconception on our analysis and results. To increase confirmability, two external evaluators reviewed our results. To increase transferability a complete description of the research process and results is provided.

### Phase two

The second phase was conducted to assess the relationship between perception of futile care, spiritual well-being, and job satisfaction among nurses in intensive care units.

#### Data gathering

Data were collected during the summer of 2017. A Census sampling method was used to select 236 nurses who were working at intensive care units of Kerman’s hospitals. Four scales were sent to all nurses within an envelope. They were asked to drop the completed questionnaires in a box in the nursing office. One hundred and eighty-seven questionnaires were returned (79% return rate).

#### Measures

The study measures were a brief demographic questionnaire, nurses’ perceptions of futile care, Minnesota Satisfaction Questionnaire (MSQ), and Spirituality and Spiritual Care Rating Scale (SSCRS). The socio-demographic questionnaire included sex, age, years of job experience, and years of work in the intensive care unit.

The nurses’ perception of futile care questionnaire has 35 items which are rated using a five-point Likert [1]. Each item scores from completely agree (4) to completely disagree (0). Higher scores indicate higher perception of futile care.

Minnesota Satisfaction Questionnaire is a twenty-item scale that measures an employee’s satisfaction with his or her job [28, 29]. Each item score on a five-point Likert from 1 (Not Satisfied) to 5 (Extremely Satisfied). Higher scores indicate higher levels of satisfaction. The Persian version of the scale was used in several studies on Iranian nurses. The internal consistency of the questionnaire in this study was (α = 0.86).

The spirituality and Spiritual Care Rating Scale is a 17-item scale that evaluates spirituality and spiritual care, religiosity, and personalized care [33]. Thirteen items scored between 1 “strongly disagree” to 5 “strongly agree,” and four items scored in reverse. Higher scores indicate a higher level of spiritual beliefs. This scale was translated into Persian and its validity and reliability were determined in previous studies. The internal consistency of the scale in this study was (α = 0.85).

#### Data analysis

Data were analyzed using SPSS 16 and AMOS 16. The correlation between job satisfaction with spiritual beliefs, age, job experience, and work experience in ICU was examined using Pearson’s correlation test. An independent t-test was used to evaluate the difference between spiritual beliefs and job satisfaction of male and female nurses. Linear regression was used to estimate the relationship between gender, spiritual beliefs, age, job experience, and work experience in ICU with job satisfaction. Structural equation modeling (SEM) was used to test the study model. The goodness-of-fit indexes were acceptable if chi-square/degree of freedom (CMIN/df) < 5, comparative fit index (CFI) > .9, standardized root mean square residual (SRMR) < .06, root mean square error of approximation (RMSEA) < .08 and Tucker– Lewis index (TLI) > .9 [34].

#### Ethical consideration

The study protocol was approved by the Ethics Committee of (omitted). Written informed consent for participation was obtained from each participant after full disclosure of the aim of the study. The participation was voluntary. We asked permission for recording the participant’s voice during the interviews.

## Results

### Phase one

The main theme of the study was a feeling of self as a useful tool in God’s hand. Sub-themes were providing care while knowing it is futile, not knowing the patient destiny, having hope to care to be fruitful, experiences patient recovery, acting to be a part of God’s plan. The main theme, subthemes, and related codes are presented in Table 2. The main themes of this study raise the question that whether there is a relationship between spiritual beliefs and job satisfaction among nurses working in the intensive care units.

**Table 2:** Subthemes and related codes of Feeling self as a useful tool in God’s hand

Main Theme	Sub-themes	Codes
Feeling self as a useful tool in God’s hand	Providing care while knowing it is futile	Doing the best, I can do Work-based on duty My duty is to care It is difficult The patient will not survive
	Not knowing the patient destiny	Do not know the future God decides about destiny What will happen is out of our control The only god knows the future What will happen is out of our knowledge Everything is possible
	Hoped to care to be fruitful	Having hope Everything we do is matter Disappointment is a sin in Islam Do not give up
	Experiences of patient recovery	There is no 100% confidence in medicine No certainty based on experience Recovery of severe cases The patient survived while we did not have hope Seeing the patient transferred to the general ward
	Acting to be a part of God plan	Being a tool in hands god Everything is a plan There is a larger image painted by god There is a reward from god Receiving reward from god

#### Feeling self as a useful tool in God’s hand

Our participants were aware of providing futile care to some patients but believing in higher powers, having a hope of patient recovery, and have faith in God were factors that help them to be more satisfied with their work. This pattern formed our main theme.

#### Providing care while knowing it is futile

Nurses were aware that some of the care provides was futile. They knew that some patients do not survive more than a few days. They expressed that providing this type of care is sometimes very difficult for them. But they believed that they must provide the care they can.

*“I know some patients will not survive; their condition is not good. It is very difficult for me, but it is not my decision, I should do my best”* (Participant 7)

*“it is the duty of the nurse to provide the best care, even he or she knows it is not effective.”* (participant 16)

#### Not knowing the patient destiny

Some nurses were defending to provide care for patients with poor prognoses. They argued that the fate of anyone is in God’s hands, and they should do their work right.

*“I do not know What will happen tomorrow if the patient dies, it is God’s will. If he or she survives also it is God’s will. No one except God knows the future. We should work based on what we know”* (Participant 13)

*“Only God determines who will live or who will die. Maybe I die today in an accident and this patient survive, Then I should do my best”* (Participant 22)

#### Hoped to care to be fruitful

Some nurses argued that they have hope that their care can be effective. They believed that their work will have a positive effect, however. Most of the participants that despite their knowledge of the poor results of their care provided it had hoped to get positive results.

*“I always have hope, every day I come to work I think about my patients to recover. I think everything I do matter; it can have positive results”* (Participant 4)

*“Disappointment is a sin in Islam. We must never give up. Every patient can get better.”* (Participant 22)

#### Experiences of patient recovery

Some nurses argued that they will do their best because they witnessed several cases recovered and discharged from ICU while they were in very bad conditions.

“Still, no physician can say with confidence that which patient will die and which one will die. We had a patient, nobody thought he would survive. He survived and I transferred him to the general ward. It was unbelievable. So, you cannot stop your caring duty.” (Participant 10)

“Our work is not based on certainty. A patient who is ill may survive. While the other patient in good condition may become infected and die. I saw many head trauma patients who transferred to general units while we thought they will die.” (Participant 5)

#### Acting to be a part of god plan

Being part of a larger plan designed by God was also considered by nurses. They discussed that they are instruments of God in a larger plan. With this worldview, they did not see their work as futile. They believed that even if their patients died, they would receive the reward for their good work.

*“We are all tools in the hands of God. Everything we do is in the plan. I always remind myself, my colleagues and students that being a nurse is a tool in God’s hands. God does it through us.”* (Participant 16)

*“When I think about the usefulness of these cares, I tell myself that even if the patient does not survive, I will receive my rewards from god*” (Participant 10)

### Phase two results

The mean ± standard deviation of age, job experience, and years of work in ICU of study subjects were 36.42 ± 8.15, 15.30 ± 8.05, and 13.20 ± 8.04 years respectively. One hundred and thirty-one (55.5%) of the study subjects were female. There was a negative correlation between age, job experience, and years of work in ICU with job satisfaction. The spiritual beliefs did not correlate with age, job experience, and years of work in ICU. The mean ± standard deviation of spiritual beliefs was 51.70 ± 11.08 and 49.96 ± 12.15 respectively in women and men (t = 1.14, df = 234, *p* = 0.252). The mean ± standard deviation of job satisfaction in women and men was 62.68 ± 12.58 and 59.11 ± 12.96, respectively (t = 2.13, df = 234, *p* = 0.03).

Multiple linear regression was calculated to predict job satisfaction based on spiritual beliefs, gender, age, job experience, years of work in ICU, and perception of futile care. A significant equation was found (f (6, 229) = 25.19, *p* < 0.001), with an R^2^ of 0.398. Gender, age, and job experience were not predictors of job satisfaction. Job satisfaction scores of nurses were equal to 21.912 + 0.493 (spiritual beliefs) – 2.474 (years of work in ICU) – 0.058 (Perception of futile care score). The linear regression is presented in Table 3.

**Table 3:** Linear regression of predictors of job satisfaction

Model		Unstandardized Coefficients		Standardized Coefficients	t	Sig.
		B	Std. Error	Beta		
1	(Constant)	21.912	15.568		1.407	.161
	SSCRS	.493	.071	.444	6.907	.000
	Gender	−2.189	1.352	−.085	−1.619	.107
	Age	1.010	.701	.641	1.441	.151
	Job experience,	1.159	1.195	.727	.969	.333
	Years of work in ICU	−2.482	.885	−1.553	− 2.804	.005
	Perception of futile care	−.058	.029	−.128	−1.997	.047

**Fig. 1:**
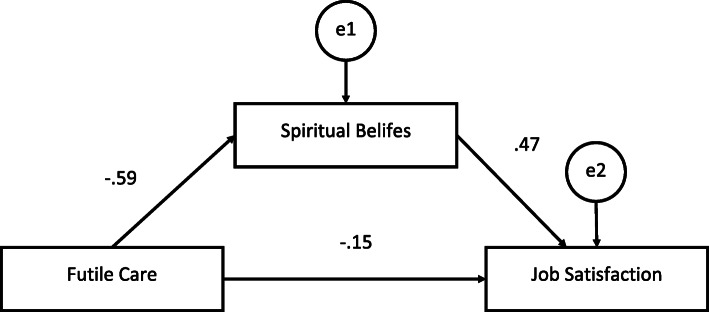
The path analysis of the moderating effect of Spiritual beliefs on the correlation of futile care perception on job satisfaction

Structural equation modeling (SEM) showed that spiritual beliefs have a moderating effect on the correlation between futile care perception and job satisfaction. The model is presented in Fig. 1. goodness-of-fit indexes were CMIN/df = 4.79, comparative fit index (CFI) =0.98, standardized root mean square residual (SRMR) = .039, root mean square error of approximation (RMSEA) = .12 and Tucker– Lewis index (TLI) = .94. The indexes showed that the model has a high goodness-of-fit. The single linear regression showed that the effect of futile care perception of job satisfaction was significant (f (1, 234) = 51.04, *p* < 0.001, R2 = 0.179, B = -0.191). Our model showed that by considering spiritual beliefs the total effect of futile care perception on job satisfaction was reduced to − 0.148.

## Discussion

This study had two phases. The first phase aimed to explore the experience of nurses in intensive care units of providing futile care with a qualitative perspective. The results of the qualitative phase raised the question of whether the spiritual beliefs of nurses who work in the intensive care units have a moderating effect on the relationship between futile care that they experience and their job satisfaction. The results of the quantitative study showed that believing in spiritual aspects of care was related to a lower sense of futile care and higher job satisfaction. Based on our results having faith and spiritual beliefs was a moderator in the relationship between experienced futile care scores and job satisfaction.

In most of the literature, scholars referred to futile care from negative aspects, which are a predictor of low job satisfaction, burnout, compassion fatigue, intention to leave, and a higher rate of turnover among nurses who work in intensive care units [35–37]. The correlation of job satisfaction with spiritual care or the spiritual well-being of nurses was assessed in previous studies [38]. For example, Khanmiri, Khodaei [39] found that there was a positive correlation between the spiritual well-being of nurses and their job satisfaction.

Our point of view toward dying and our moral perspective regarding the results of our actions may affect our sense of futility and satisfaction with our job [40]. This point of view raises mostly from individual moral standards which we can explain based on moral theories. There are some moral theories including utilitarianism, Kantianism, virtue theory, the four principles approach (Principlism) which we can use to explain moral standards [41, 42]. The most acceptable and used theory in medicine is the four principles approach. Based on this theory moral decisions in medicine can be made according to four commonly accepted principles of patient autonomy, non-maleficence, beneficence, and justice [42, 43]. This theory has an easier practical clinical application than other theories [43]. When a nurse provides care for patients with a low expectation of recovery, it can cause an experience of futile care. Based on our results, nurses with spiritual believes see their works in line with God’s will and they try to respect the patient’s autonomy, provide non-maleficence, and beneficence care along with justice for all patients. Working based on these principles may reduce the moral distress of nurses. The results of previous studies showed that moral distress was related to low job satisfaction and intention to leave in nurses [16, 44]. Spiritual well-being can decrease the dissatisfaction related to moral distress. Spiritual care is a way to decrease the sense of futility. However, the results of a literature review showed that there is a need to improve knowledge and skills regarding the spiritual care of ICU healthcare professionals through relevant training courses [45].

Spiritual aspects should be considered in the work environment of nurses. It is necessary for compassionate caring. It is argued by Paal, Neenan [46] that there is a motivation to change the culture of health care organizations to be more compassionate and caring. The change in the workplace and using compassionate leadership is an essential factor. They suggested spiritual leadership as an emergent solution to transform the healthcare workplace.

### Limitations

Futile care is a multidimensional concept. Several factors can affect the experience of futile care. Despite the limitation that we have to include all of the effective factors, we tried to include some variables including the spiritual believes of nurses. Future studies can assess the effect of other variables. Another limitation of the study was related to the scales. We used a general scale for spiritual well-being. The definition of spirituality is different in different contexts and its relationship with religion is very strong. We suggest using more specific scales in future studies.

## Conclusions

Nursing is a caring profession. Providing care for patients with poor outcomes can cause a sense of futility in nurses. Our results showed that futile care can decrease the job satisfaction of nurses. Also, nurses who have higher spiritual well-being had higher job satisfaction and lover sense of providing provide care. Spiritual well-being had a moderating effect on the relationship between futile care and job satisfaction among nurses.

Health care managers should consider spiritual aspects of care as important parts of health services. Nurses’ spiritual well-being can affect their job satisfaction. It can also affect the care they provide. By improving the spiritual health of nurses, their job satisfaction and quality of care can be enhanced. Spiritual health can be enhanced by providing spiritual resources in the workplace, such as facilitating religious observances. Providing spiritual care to patients is another thing that can be done. Teaching the importance of paying attention to the spiritual aspects of care should be considered by nursing managers. One implication for our results is changing workplace spiritual climate to reach better job satisfaction for nurses and helping to provide quality care for patients.

## Data Availability

The datasets used and/or analyzed during the current study are available from the corresponding author on reasonable request.

## Abbreviations

MSQ: Minnesota Satisfaction Questionnaire
SSCRS: Spirituality and Spiritual Care Rating Scale
